# Validation of an automated premature ventricular contraction mapping algorithm

**DOI:** 10.1007/s10840-025-02150-4

**Published:** 2025-10-21

**Authors:** Yuji Ishida, Michael R. Gold, Joshua E. Payne, Adam R. Bainey, Michael E. Field, Patrick Badertscher, Jeffrey R. Winterfield

**Affiliations:** 1 Division of Cardiology, Medical University of South Carolina, 30 Courentay Drive, MSC 592, Charleston, SC 29425-5920 USA; 2 Department of Cardiology, Hirosaki University Graduate School of Medicine, Hirosaki, Aomori, Japan

**Keywords:** Catheter ablation, Cardiac mapping, Ventricular arrhythmia, PVC

## Abstract

**Background:**

Catheter mapping and ablation of premature ventricular contractions (PVCs) requires accurate annotation of earliest local activation time (LAT), but displacement in catheter position between sinus rhythm (SR) and the PVC complicates three-dimensional mapping localization. An automated algorithm to annotate LAT sites at the corresponding sinus rhythm sites would provide an alternative to manual annotation and improve procedural efficiency.

**Methods:**

A retrospective single center study assessed 64 patients undergoing catheter ablation of PVCs. We divided the study patients into two groups: the patients underwent RF ablation using the CARTO 3 version 7 with LAT-hybrid™ module (hybrid group) and the other patients using the CARTO 3 version 6 without LAT-hybrid™ module (conventional group).

**Results:**

The primary results of this study demonstrate that the mean hybrid distance is 4.03 ± 2.33 mm, and this automated algorithm can correct for the positional shift with accuracy comparable to manual correction. Moreover, this algorithm showed significantly shorter RF time and the shorter PVC offset compared to the conventional method.

**Conclusion:**

PVC mapping with the LAT-hybrid™ module demonstrated that an automated algorithm can map in more detail with no compromise in accuracy. The LAT-hybrid™ module showed significantly shorter RF time and PVC offset from the earliest LAT in LVOT cases.

**Graphical Abstract:**

Spatial displacement of earliest site of activation for premature ventricular contractions (PVCs) relative to sinus rhythm beats in high-risk para-His bundle region. Automated spatial correction (LAT-Hybrid) provides a more focal area of interest 1.4mm posterior to the His region adjacent to the tricuspid annulus. The LAT-Hybrid automated algorithm directed ablation to the successful site with single burn elimination.

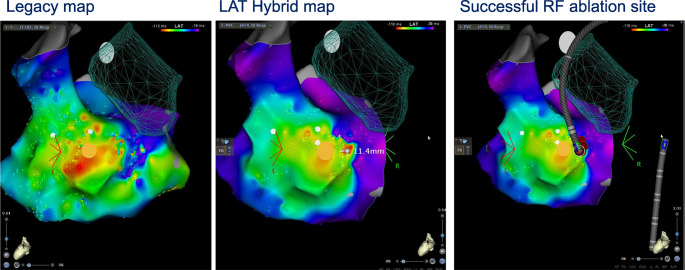

## Introduction

Catheter ablation using electroanatomic mapping (EAM) is an established treatment for patients with premature ventricular contractions (PVCs) [1, 2]. However, the EAM guidance of this procedure is complicated by the spatial displacement between catheter positions in sinus rhythm (SR) compared with during a PVC [3–5]. When ablating, there is an inherent challenge to direct the catheter to the earliest PVC activation time. The traditional method used is to perform a manual annotation of PVC local activation time (LAT) and compare with that same catheter position in SR. This can then be used to correct the spatial shift, but it adds significant time to the procedure and may vary by PVC location.

Automated algorithms have been developed to correct for the spatial shift associated with PVCs. LAT-hybrid™ module (CARTO 3 version 7, Biosense Webster, Diamond Bar, CA, USA) generates a hybrid map with PVC LAT points placed on SR EAM. The resulting hybrid map prevents need for toggling between a map of sinus rhythm points and the separate PVC LAT map. Distances are calculated between the correlated sinus rhythm and PVC locations. There are limited data on the effectiveness of this system. Accordingly, we present a retrospective study designed to assess the accuracy and efficiency of the LAT- hybrid™ module during PVC ablation.

## Methods

We performed a retrospective study of patients undergoing PVC ablation. This study was approved by the institutional review board, and all patients gave informed consent for catheter ablation.

### Study population

From November 2018 to February 2020, 64 patients underwent PVC ablation at the Medical University of South Carolina for standard indications according to current guidelines [1, 2]. Thirty-eight patients with PVCs who underwent successful radiofrequency (RF) ablation using the CARTO 3 module were included in the present study. The remaining 26 patients were excluded for the following reasons: alternative EAM system was used (n = 10), pace mapping rather than activation mapping was the procedural approach (n = 8), insufficient data were available (n = 4), the procedure was only partially successful (n = 3), and the cryoablation was used in addition to radiofrequency ablation (n = 1).

We divided the study patients into two groups: the patients underwent RF ablation using the CARTO 3 version 7 with LAT-hybrid™ module (hybrid group) and the other patients using the CARTO 3 version 6 without LAT-hybrid™ module (conventional group).

### LAT-hybrid™ module

**Fig. 1:**
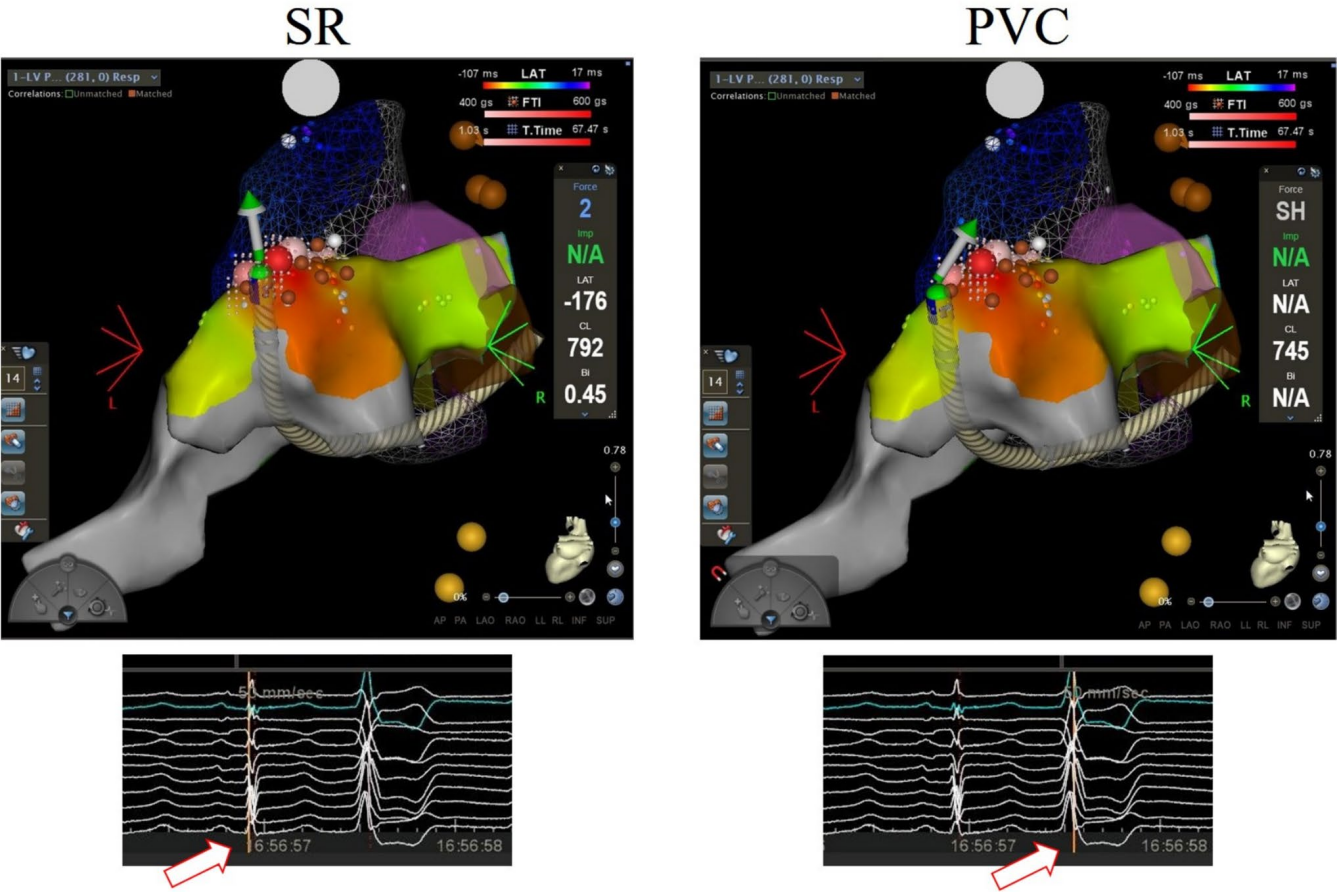
The spatial displacement between catheter positions in sinus rhythm (SR) and a premature ventricular contraction (PVC). LAT value is taken from the PVC beats (right panel). Point location is taken from the earliest SR beats preceding the PVC (left panel). Arrows indicated the timing of annotation

A LAT-hybrid™ module is a novel tool for mapping PVC added in CARTO 3 version 7. This module will have an SR anatomy with PVC activation. When PVC is detected, two beats are selected for the acquisition. LAT value is taken from the PVC beats. Point location is taken from the immediate SR beat preceding the PVC (i.e. R-wave peak of PVC and R-wave peak of the previous SR) (Fig. 1). The resulting ‘LAT-hybrid’ activation map provides PVC LAT annotation on the corresponding 3-D anatomic site in sinus rhythm. The distance of catheter displacement between PVC to SR (hybrid distance) is automatically calculated by this module.

### Electrophysiological study and ablation

All patients underwent an electrophysiological study before RF ablation to identify the origin of PVC. Antiarrhythmic drugs and beta-blockers were withdrawn for five half-lives before the procedure. During the procedure, 12-surface ECG and intracardiac recordings were displayed by an electrophysiology data acquisition system (CaridoLabo, GE Healthcare, Houston, TX, USA).

The DECANAV (Biosense Webster), the PENTARAY (Biosense Webster), the NogaStar (Biosense Webster), and/or the Thermocool Smarttouch SF (Biosense Webster) was used to obtain an activation map of the right ventricle (RV), the left ventricle (LV), or the coronary sinus (CS) at the discretion of the physician.

Point-by-point mapping was performed during PVCs using the Wavefront mapping annotation and the Advanced Reference Annotation Criterion: Automatic. During continuous mapping, the nominal setting of pattern matching thresholds for activation (i.e. PVC) and location (i.e. SR) were 0.90 and 0.96, respectively. These parameters were changed at the discretion of the physician. Points were acquired only if the pattern matching score was greater than the threshold. Position stability was 4 mm and LAT stability 3 ms.

Intravenous sedation was minimized to avoid anesthesia-related PVC suppression. Isoproterenol or epinephrine were used to induce PVCs in cases where spontaneous PVCs were otherwise too infrequent for activation mapping. These agents were used routinely to assess inducibility postprocedure.

We delivered RF ablation at the earliest LAT point, prepotential, and/or optimal pace map using an open-irrigated ablation catheter (Thermocool Smarttouch SF, Biosense Webster). Ablation power and duration were performed at the discretion of the operator in the power control mode with starting power 30-35W. The site of successful ablation was retrospectively identified. Acute success was defined as elimination of target PVCs with radiofrequency ablation after sufficient waiting period and after challenge with isoproterenol, epinenphrine, and/or burst pacing from the right ventricle.

### Hybrid distance analysis

In the hybrid group, the hybrid distance was collected from the point list. Variables that potentially influence hybrid distance were also taken into account: (1) the mapping location (the RV, the LV, and the CS), (2) the type of mapping catheter (bipolar [the NogaStar and the Thermocool Smarttouch SF] or multipolar [the DECANAV and the PENTARAY]), and (3) the PVC coupling interval.

### Procedure parameters analysis

**Fig. 2:**
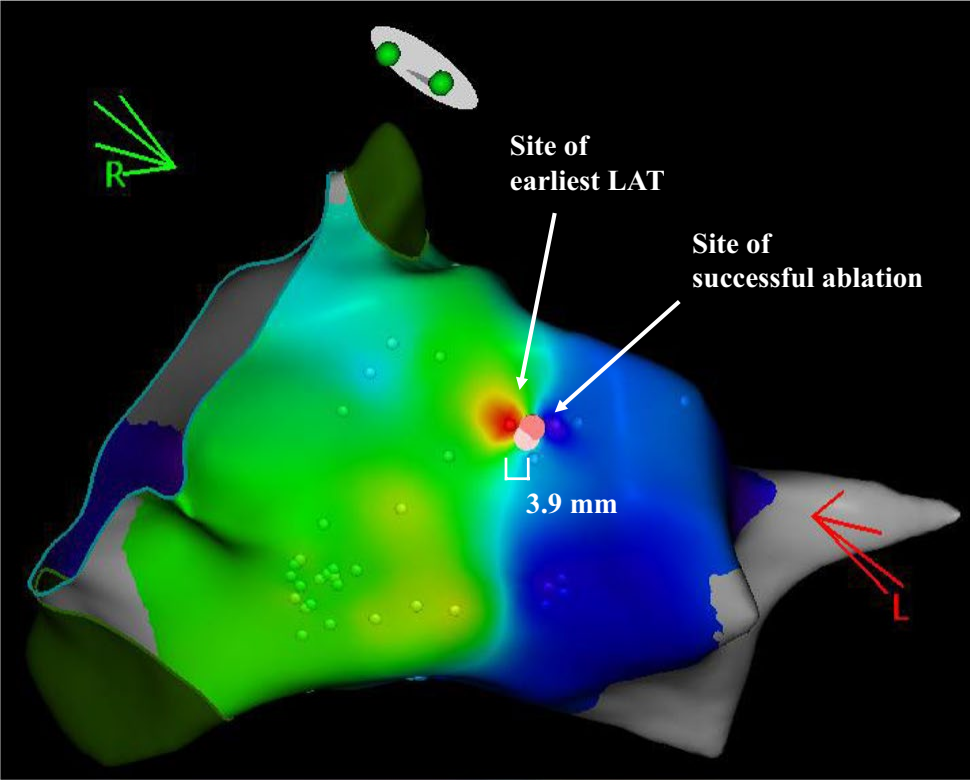
Comparison of the earliest local timing activation (LAT) with the successful ablation site. The site of the earliest LAT of PVC (red spot) is far (3.9 mm) from the site of successful ablation (dark pink tag). This is the representative case of PVC originating from left ventricular summit

The procedure parameters including RF time, the number of RF applications were collected. The distance between the site of successful ablation and the earliest LAT of the PVC (PVC offset from the earliest LAT) were measured (Fig. 2). When a prepotential was recorded, we also measured the distance between the site of successful ablation and the site of the prepotential (PVC offset from the prepotential). We compared these parameters between the two groups. Additionally, we analyzed these parameters by the chamber or the PVC origin when there were over three cases in each group.

### Statistical analysis

Continuous variables are presented as mean ± standard deviation and were compared using Student’s t-test. Categorical variables are summarized as frequencies and percentages and compared using Fisher’s exact test. Mean hybrid distance according to the location and the type of the catheter for each patient was analyzed by using the paired t-test. Regression analysis was used to assess the relationship between the mean hybrid distance and the mean coupling interval. The statistical analysis was performed using JMP 13.2.1 (SAS, Cary, NC, USA) and a p-value < 0.05 was considered statically significant.

## Results

### Patient characteristics

We included 19 patients with 21 PVCs in the hybrid group and 19 patients with 19 PVCs in the conventional group. The patient characteristics of the hybrid and conventional groups are presented in Table 1. Patients in the hybrid group were more likely to take beta-blocker (84% vs 47%, P = 0.038). There were no differences in other parameters between the two groups (Table 1).

**Table 1:** Comparison of baseline characteristics between the hybrid group and the conventional group

	Hybrid group (N = 19)	Conventional group (N = 19)	P-value
Age (years)	69 ± 9	63 ± 12	0.10
Male gender	12 (63)	13 (68)	1.00
BMI (kg/m^2^)	28.0 ± 4.1	29.2 ± 7.3	0.55
Hypertension	10 (53)	11 (58)	1.00
Diabetes Mellitus	4 (21)	1 (5)	0.34
Atrial fibrillation	4 (21)	1 (5)	0.34
Cardiomyopathy	10 (53)	4 (21)	0.09
Presence of ICD	4 (21)	3 (16)	1.00
LVEF	50 ± 17	53 ± 13	0.49
Medication			
Beta-blocker	16 (84)	9 (47)	0.038
ACE/ARB or ARNI	12 (63)	7 (37)	0.19
Class I AAD	4 (21)	3 (16)	1.00
Class III AAD	3 (16)	3 (16)	1.00
Creatine (mg/dl)	1.12 ± 0.28	1.12 ± 0.60	0.99
Hemoglobin (g/dl)	14.8 ± 1.5	15.5 ± 1.3	0.11

Values are presented as mean ± standard deviation or as n (%). AAD, antiarrhythmic drug; ACE, angiotensin converting enzyme inhibitor; ARB, angiotensin II receptor blocker; ARNI, angiotensin receptor neprilysin inhibitor; BMI, body mass index; ICD, implantable cardioverter-defibrillator; LVEF, left ventricular ejection fraction

### Hybrid distance

In the hybrid group, a total of 4955 points from 46 maps were analyzed. This included 14 maps and 1654 points from the RV, 20 maps and 2742 points from the LV and 12 maps and 559 points from the CS. The mean hybrid distance for all points was 4.03 ± 2.33 mm.

**Fig. 3:**
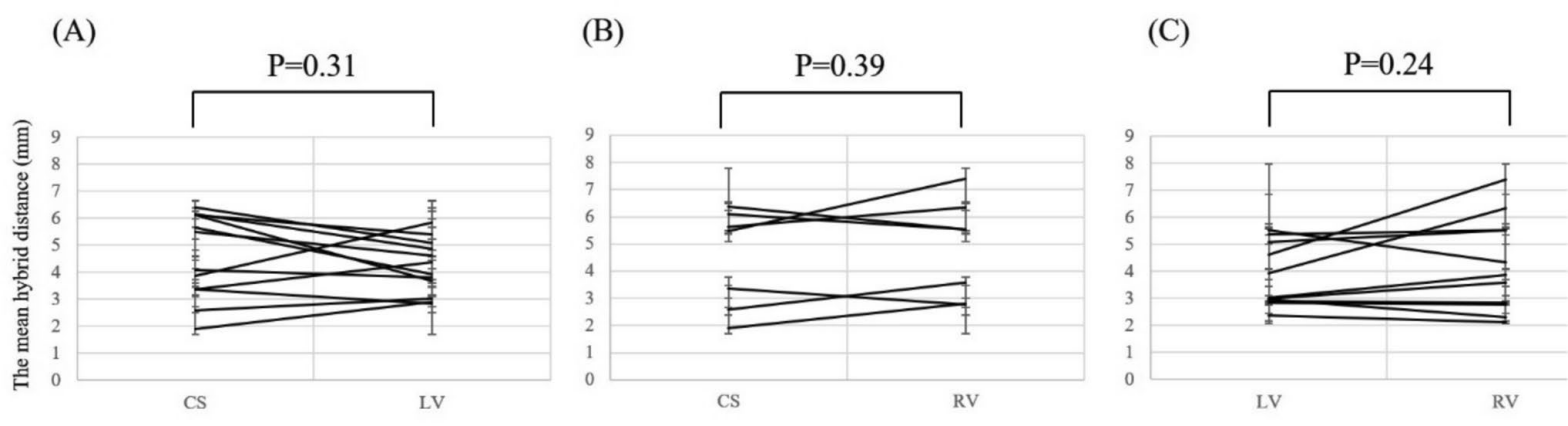
Analysis of the mean hybrid distance according to the location for each patient. Panel A: Comparison of the mean hybrid distance from the coronary sinus (CS) and the left ventricle (LV). Panel B: Comparison of the mean hybrid distance from the CS and the right ventricle (RV). Panel C: Comparison of the mean hybrid distance from the LV and the RV. There were no statistical differences between the mean hybrid distance and the location

**Fig. 4:**
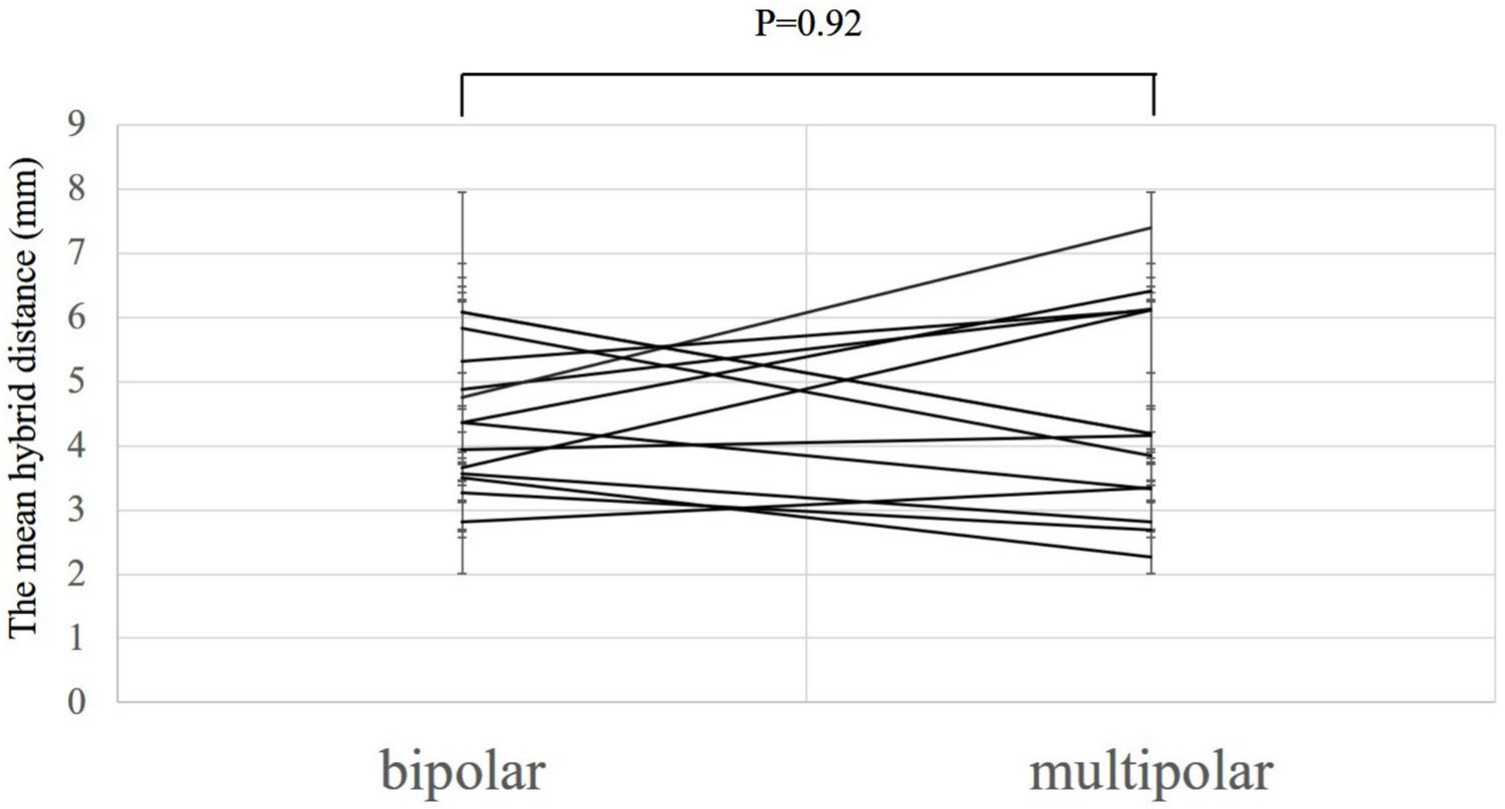
Analysis of the mean hybrid distance according to the type of catheter. There were no statistical differences between the mean hybrid distance and the type of the catheter

**Fig. 5:**
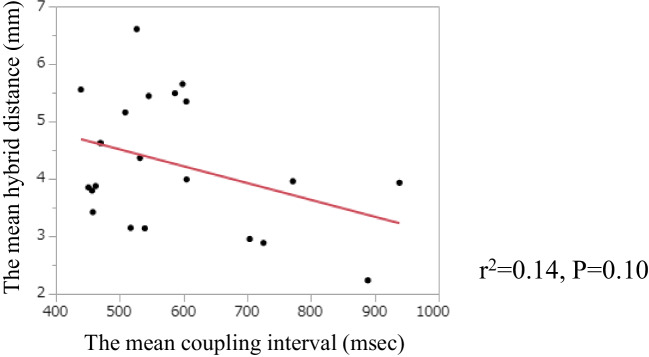
Regression analysis of the mean hybrid distance vs the mean coupling interval. There was no statistical correlation between the mean hybrid distance and the mean coupling interval

There were no differences in the mean hybrid distances between the location for each patient (LV 4.19 mm vs CS 4.58 mm, n = 12, p = 0.45; RV 4.86 mm vs CS 4.49 mm, n = 7, p = 0.39; and RV 4.24 mm vs LV 3.77 mm, n = 11, p = 0.24, respectively) (Fig. 3). Fourteen PVCs were mapped by both the bipolar and the multipolar catheter, whereas the other 7 PVCs were mapped only by the bipolar catheter. There were also no significant differences between the type of catheter (bipolar 4.50 mm vs multipolar 4.60 mm, P = 0.92) (Fig. 4). Moreover, there was no correlation between the mean hybrid distance and the mean PVC coupling interval for each patient (r^2^ = 0.14, P = 0.10) (Fig. 5).

### Comparison in the procedure parameters between the hybrid group and the conventional group

The most common origin of PVCs in this series was the LV outflow tract (LVOT) (n = 15) including aortic sinus of Valsalva, LV summit and subvalvular LVOT. The next most common PVC origin was the RV outflow tract (RVOT) (n = 8) in this cohort. Eleven patients have recorded prepotential in the hybrid group compared with 9 patients in the conventional group (Table 2). For the overall study population, we observed no statistical differences in the number of points/map, RF time, or the number of RF applications between the hybrid group and the conventional group. Moreover, there were no statistical differences in the PVC offset between the two groups (Table 3). However, in only the LVOT cases, RF time and PVC offset from the earliest LAT appeared significantly reduced in the hybrid group compared with the conventional group (215 ± 191 vs 685 ± 544 s, P = 0.049; 5.74 ± 4.80 vs 12.31 ± 6.42 mm, P = 0.045, respectively) (Table 4).

**Table 2:** Patient-level data of procedure

group	Age	sex	Chamber	Origin of PVC	Procedure parameters				
					Number of points	RF time	Number of RF applications	PVC offset from the earliest LAT	PVC offset from the prepotential
hybrid	71	M	RV, LV	peri His	547	605	14	5.2	
hybrid	69	F	RV, LV	Intramural OT	101	539	10	10.4	6
hybrid	72	F	RV, LV, CS	ASOV (LCC)	382	120	2	1.9	
hybrid	76	M	LV, CS	PM in LV	117	313	4	9.5	16.2
hybrid	75	M	RV, LV, CS	ASOV (LCC)	216	114	2	5	
hybrid	62	F	LV, CS	LV summit	55	251	5	15.2	10.4
hybrid	72	M	LV, LV remap	MVA	266	928	11	4.3	8.1
hybrid	69	F	RV, LV, LV remap, CS	LV summit	463	169	3	3.9	6.6
hybrid	57	M	RV, LV, CS	Intramural OT	341	393	8	8	
hybrid	82	M	RV, LV, CS	Subvalvular LVOT	767	632	13	4.1	
hybrid			RV, LV	PM in LV	348	421	9	10.1	
hybrid	61	F	RV, LV	RVOT	485	837	6	11.8	15.3
hybrid			RV	TVA	30	349	11	13.9	9.1
hybrid	58	M	LV	TVA	20	149	2	11	4.9
hybrid	58	M	LV, CS	MVA	35	740	12	9.5	4.6
hybrid	86	M	RV	PM in RV	77	113	3	4.1	
hybrid	56	M	RV, LV, CS	ASOV (RCC)	138	100	2	8.7	
hybrid	58	F	RV	RVOT	137	176	10	0	
hybrid	81	M	LV, CS	LV summit	44	119	1	1.4	
hybrid	63	M	LV, CS	MVA	277	211	4	7.2	8.9
hybrid	77	F	RV, LV, CS	RVOT	109	306	5	1.0	8.9
conventional	36	F	RV, LV, CS	RVOT	195	493	9	11.9	11.8
conventional	76	F	RV, LV	ASOV (RL commissure)	114	175	5	7.6	6.8
conventional	64	M	RV, LV, LV remap, CS	LV summit	97	932	13	13.5	16.7
conventional	72	F	RV, LV, CS	ASOV (LCC)	42	443	6	19.8	
conventional	56	F	RV, LV	LV summit	161	522	4	6.8	5.3
conventional	47	F	LV	PM in LV	192	514	3	12.1	
conventional	44	M	RV	RVOT	164	486	9	10.5	
conventional	68	M	RV, LV	Crux	261	554	16	7.2	5.7
conventional	80	M	RV, LV	Intramural OT	80	506	6	8.9	
conventional	71	M	RV, LV, CS	ASOV (RCC)	122	303	14	22.1	
conventional	60	M	RV, LV, CS	ASOV (RL commissure)	478	1871	35	3.3	
conventional	60	M	RV, LV, CS	Intramural OT	74	210	4	3.9	3.4
conventional	53	F	RV	RVOT	28	49	4	3.5	
conventional	63	M	RV, CS	TVA	56	268	8	4.9	5.3
conventional	54	M	RV, LV, CS	MVA	153	372	6	6.4	
conventional	84	M	RV	RVOT	105	207	6	8.3	
conventional	64	M	LV, CS	ASOV (RL commissure)	259	390	4	13.3	15
conventional	67	M	RV, RV remap, LV, CS	ASOV (RL commissure)	471	840	14	12.1	11.9
conventional	70	M	LV	PM in LV	164	180	1	4.1	

ASOV, aortic sinus of Valsalva; CS, coronary sinus; LAT, local activation time; LCC, left coronary cusp; LV, left ventricular; MVA mitral valve annulus; OT, outflow tract; PVC, premature ventricular contraction; PM, papillary muscle; RCC, right coronary cusp; RF, radiofrequency; RL, right coronary cusp- left coronary cusp; RV, right ventricular; TVA, tricuspid valve annulus

**Table 3:** Comparison of the procedure parameters in all cases (N = 40) between the hybrid group and the conventional group

	Hybrid group N = 21	Conventional group N = 19	P- value
Number of points	236 ± 204	167 ± 126	0.21
Number of maps	2.2 ± 0.8	2.3 ± 1.0	0.8
Number of points/maps	103 ± 80	84 ± 58	0.39
RF time (sec)	369 ± 252	490 ± 401	0.25
Number of RF applications	6.5 ± 4.2	8.8 ± 7.6	0.27
PVC offset from the earliest LAT (mm)	6.96 ± 4.33	9.48 ± 5.27	0.1
PVC offset from the pre-potential (mm) Hybrid N = 11, Conventional N = 9	9.00 ± 3.81	9.09 ± 4.84	0.96

LAT, local activation time; RF, radiofrequency; PVC, premature ventricular contraction

**Table 4:** Comparison of the procedure parameters in left ventricular outflow tract cases (N = 15) between the hybrid group and the conventional group

	Hybrid group N = 7	Conventional group N = 8	P- value
Number of points	296 ± 261	214 ± 172	0.48
Number of maps	2.9 ± 0.7	2.9 ± 0.8	0.97
Number of points/maps	96 ± 82	76 ± 53	0.58
RF time (sec)	215 ± 191	685 ± 544	0.049
Number of RF applications	4.0 ± 4.1	11.9 ± 10.4	0.08
PVC offset from the earliest LAT (mm)	5.74 ± 4.80	12.31 ± 6.42	0.045
PVC offset from the pre-potential (mm) Hybrid N = 2, Conventional N = 5	8.50 ± 2.69	11.14 ± 4.98	0.52

LAT, local activation time; RF, radiofrequency; PVC, premature ventricular contraction

## Discussion

### Major findings

To our knowledge, this is the first clinical study that investigated the accuracy of the LAT-hybrid™ module. The primary results of this study demonstrate that the mean hybrid distance is 4.03 ± 2.33 mm, and this automated algorithm can correct for the positional shift with accuracy comparable to manual correction. Moreover, this algorithm showed significantly shorter RF time and shorter PVC offset compared to the conventional method for PVCs originating from the LVOT.

### Comparison with previous studies

Previous studies that corrected the manual position shift showed the displacement between the point position in SR and PVC was about 4 to 9 mm. [3, 4] Andreu et al. demonstrated that this displacement was greater in RV than in LV, and a shorter coupling interval of the PVC was associated with greater displacement [3]. Our study showed the spatial displacement correcting automated algorithm is similar to or shorter than the manual method. However, this displacement was not influenced by the chamber or the coupling interval in our study. The reasons for these differences are not known. The greater displacement with shorter coupling interval may be amplified in patients with hyperdynamic ventricular systolic function and dampened in patients with depressed systolic function [4]. However, the mean LV ejection fraction is smaller in our study than in the Andreu et al. study. They also mentioned the observation that PVCs originating in the LV had a longer coupling interval could at least partially explain them. In this regard, there were no differences in the coupling interval between the left side of the origin and the right side one (607 ± 130 vs 519 ± 122, P = 0.39).

Recently, dePotter et al. showed that LAT hybrid mapping demonstrated a high and acute and long-term success rate of PVC ablation [5]. The position shift between the best pacemap and hybrid earliest LAT position was 7 mm closer to the position shift of conventional LAT point in this study. This mapping system was developed specifically for this study. The concept of this mapping system was the same as the LAT hybrid™ module. However, the mean spatial displacement was 8.9 mm in their study. This displacement was more than double compared to our results. One possible explanation was that the average number of the acquisition point per PVC of their study was smaller than that of ours (7 vs 236).

### Mapping and ablation of LVOT origin PVCs

There were no statistical differences in the PVC offset from the earliest LAT between the hybrid group and the conventional group in all cases. However, we identified that the LAT-hybrid module™ shortened RF time and PVC offset form the earliest LAT compared to the conventional method. We thought the larger the hybrid distance, the greater the shift correction by this module, and the more accurate ablation could be achieved. However, there were no statistical differences in the mean hybrid distance between the LVOT origin and the non-LVOT origin (4.69 ± 1.38 vs 4.04 ± 0.98 mm, P = 0.22). Thus, it is unclear why this result was obtained only in LVOT cases.

### Clinical implication

The CARTO LAT-hybrid™ module provides an easy and automated method to address spatial shifts between sinus rhythm and PVCs which may improve mapping accuracy and permit more efficient navigation to target ablation sites during sinus rhythm. Moreover this new system enables operators to avoid manual adjustment of points between sinus rhythm and PVCs to address shifts in a more conventional mapping setting. Elimination of human input into map adjustments may provide more rapid and reproducible maps less prone to error from spatial shifts. Based on our data, this module may be most useful for LVOT origin PVC mapping and ablation.

The dataset incorporates ablation cases performed from 2018–2020 using lower density catheters compared to more recent additions including the Optrell™ and Octaray™ catheters. The LAT-hybrid™ would remain effective with these newer catheters, and the use of older generation catheters for the cases described in this manuscript would not affect the overall findings or conclusions.

### Study limitations

There were several limitations to our study. First, this was a relatively small, single-center, retrospective study, and physician operators remained unblinded with conventional vs LAT-hybrid™ module utilization during mapping. Importantly also, we did not provide long-term outcome of PVC ablation. Further prospective randomized studies with operators blinded to mapping algorithm may be helpful to determine if the LAT-hybrid™ module improves outcomes acutely and with long term suppression of target PVCs. Additionally, the dataset fails to include long term follow up data post-ablation due to inconsistent follow up of the cohort given the referral nature of the study population.

## Conclusion

The initial clinical experience of PVC mapping with the LAT-hybrid™ module demonstrated that an automated algorithm provides rapid mapping without compromise in accuracy. In cases involving PVCs from the LVOT, the LAT-hybrid™ module yielded significantly shorter RF time and PVC offset from the earliest LAT in LVOT cases.
